# Crystal structure and Hirshfeld surface analysis of the organic–inorganic hybrid compound tris­(2-iodo­ethyl­ammonium) hexa­iodido­bis­muthate(III)

**DOI:** 10.1107/S2056989025000386

**Published:** 2025-01-24

**Authors:** Olesia I. Kucheriv, Valeriia M. Ovdenko, Iryna S. Kuzevanova, Irina A. Golenya, Il’ya A. Gural’skiy

**Affiliations:** a Department of Chemistry, Taras Shevchenko National University of Kyiv, Volodymyrska St. 64, Kyiv 01601, Ukraine; b Department of General and Inorganic Chemistry, National Technical University of Ukraine "Igor Sikorsky Kyiv Polytechnic Institute", Beresteiskyi Pr. 37, 03056 Kyiv, Ukraine; Vienna University of Technology, Austria

**Keywords:** crystal structure, bis­muth(III) iodide, organic cation, hybrid material

## Abstract

The asymmetric unit of the title organic–inorganic salt, (IC_2_H_4_NH_3_)_3_[BiI_6_], consists of a [BiI_6_]^3−^ octa­hedron and three 2-iodo­ethyl­ammonium cations. The octa­hedra do not inter­act with each other, and the supra­molecular arrangement is ensured by an intricate network of N—H⋯I hydrogen bonds between cations and anions. In addition, a weak I⋯I inter­action between an organic cation and the coordination octa­hedron is present in the crystal structure. Hirshfeld surface analysis revealed that the most important contributions to the crystal packing are from H⋯I (72.3%) and I⋯I (11.3%) inter­actions.

## Abstract 2

The crystal structure of (IC_2_H_4_NH_3_)^+^_3_[BiI_6_]^3−^ is composed of discrete [BiI_6_]^3–^ octa­hedra, the charge of which is compensated by three crystallographically independent 2-iodo­ethyl­ammonium cations.

## 1. Chemical context

Organic–inorganic halidobismuthates(III) represent a promising class of hybrid compounds that attract attention due to their structural versatility in combination with inter­esting physical properties. For example, these bis­muth-based compounds are currently used as a less toxic alternative to lead-based perovskites for applications as light-emitting diodes (Zhou *et al.*, 2018 ▸), for X-ray detection (Wang *et al.*, 2023 ▸) or for photovoltaics (Zhang *et al.*, 2020 ▸).

Hybrid halidobismuthates(III) contain the [Bi*X*_6_]^3–^ (*X* = Cl, Br or I) coordination octa­hedron as a fundamental building block, which can assemble into very different topologies in the crystal structure, starting from those containing discrete anionic halidometallic units up to structures with anionic chains or layers (alternatively named as 0-D, 1-D and 2-D halidometallic building blocks). For instance, hybrid bis­muthates with general formula *A*_3_[Bi_2_*X*_9_] tend to crystallize with two structural set-ups: one forms inorganic layers, exemplified by methyl­ammonium (MA) bis­muth bromide (MA)_3_[Bi_2_Br_9_] (Li *et al.*, 2019 ▸), while the other one is characterized by a formation of isolated face-sharing [Bi_2_*X*_9_]^3–^ bi-octa­hedra, exemplified by (MA)_3_Bi_2_I_9_ (Hoye *et al.*, 2016 ▸).

Inter­estingly, the decrease of dimensionality in the anion leads to an increased localization of electronic states and decreased valence and conduction bands, which results in the occurrence of self-trapped excitons and strong excitonic emission. Hence, highly effective luminescence with different emission wavelengths have been observed for 0-D halidobismuthates. For example, highly efficient blue (480 nm) emission with a quantum yield of 58% was achieved for 0-D hybrid tetra­phenyl­phospho­nium (TPP) bis­muth chloride (TPP)_2_[BiCl_5_] (Lai *et al.*, 2024 ▸). At the same time, 4-(chloro­meth­yl)pyridinium bis­muth chloride (4-cmpyH)_2_[BiCl_5_] was shown to display yellow luminescence with an emission wavelength of 597 nm and a quantum yield of 5.56% (Qi *et al.*, 2022 ▸).
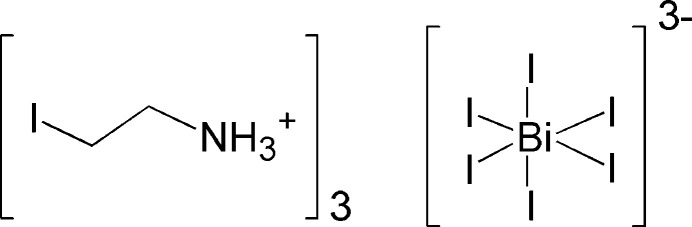


In this context, we report here on the crystal structure and Hirshfeld surface analysis of a new organic-inorganic compound, (2-iodo­ethyl­ammonium)_3_[BiI_6_], which is composed of discrete [BiI_6_]^3–^ anions.

## 2. Structural commentary

**Figure 1:**
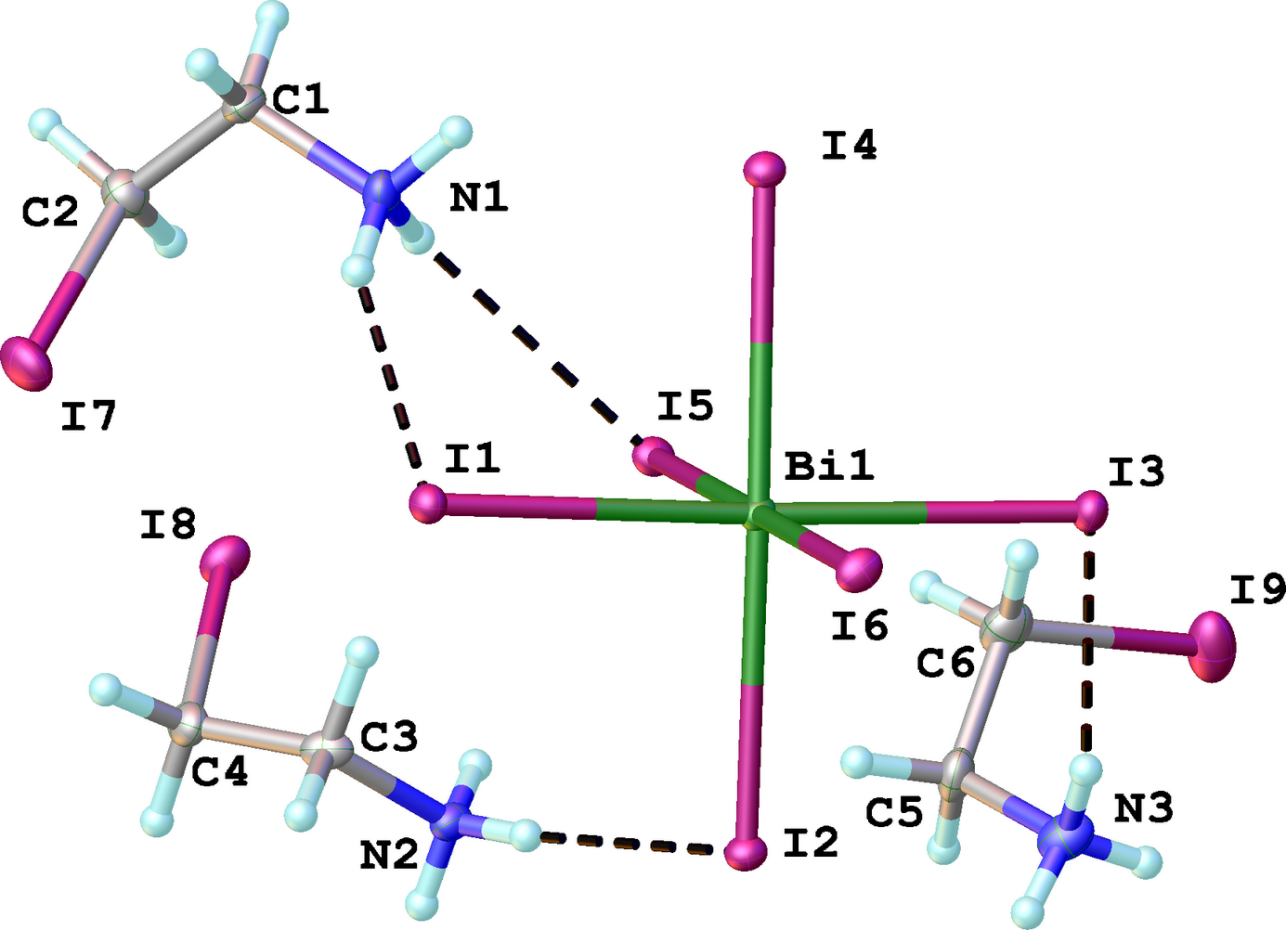
The mol­ecular structures of the entities in the asymmetric unit of the title compound. Displacement ellipsoids are drawn at the 50% probability level; dashed lines represent N—H⋯I hydrogen bonds.

**Figure 2:**
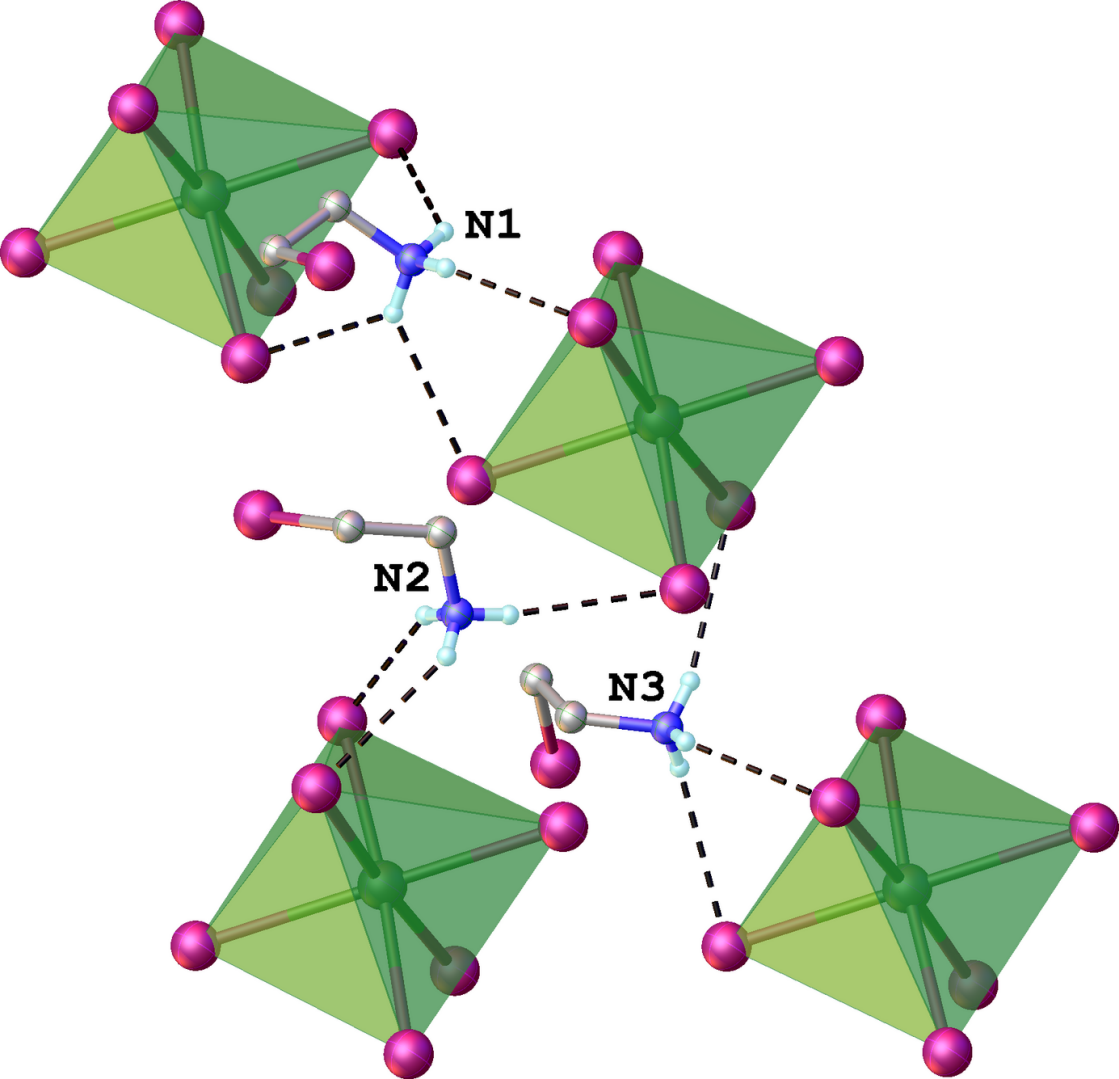
Details of the N—H⋯I hydrogen-bonding network (dashed lines) between organic cations and inorganic anions (represented as polyhedra). Only H atoms involved in these inter­actions are shown.

The coordination octa­hedron [BiI_6_]^3–^ is slightly distorted (Fig. 1 ▸), with Bi—I bond lengths ranging from 3.0287 (3) to 3.1333 (3) Å (Table 1 ▸). Such a small variation in bond length leads to a relatively small bond length distortion parameter, Δ*d* = 1/6 Σ(*d_i_* − *d*)^2^/*d*^2^ = 1.1·10^−4^ (where *d_i_* is one of six individual bond lengths in the octa­hedron and *d* is the mean Bi—I bond length). The *cis*-(I—Bi—I) angles (*α*) lie in the inter­val 88.529 (9)–91.561 (9)°, which also indicates the occurrence of octa­hedral distortion, and can be described by the following parameter, Σ = Σ|90°–α| = 19.674°. The formed coordination octa­hedra are isolated, providing a 0-D topology in the crystal structure (Fig. 2 ▸); these octa­hedra are aligned parallel to (003). The charge of the [BiI_6_]^3–^ anions is balanced by three crystallographically unique 2-iodo­ethyl­ammonium cations, the I—C, C—C and C—N bond lengths of which are within the expected range. All three 2-iodo­ethyl­ammonium cations adopt a synclinal conformation with torsion angles of 68.6 (5)° (for the N1-containing cation), −66.2 (4)° (N2) and 64.1 (5)° (N3).

## 3. Supra­molecular features

**Figure 3:**
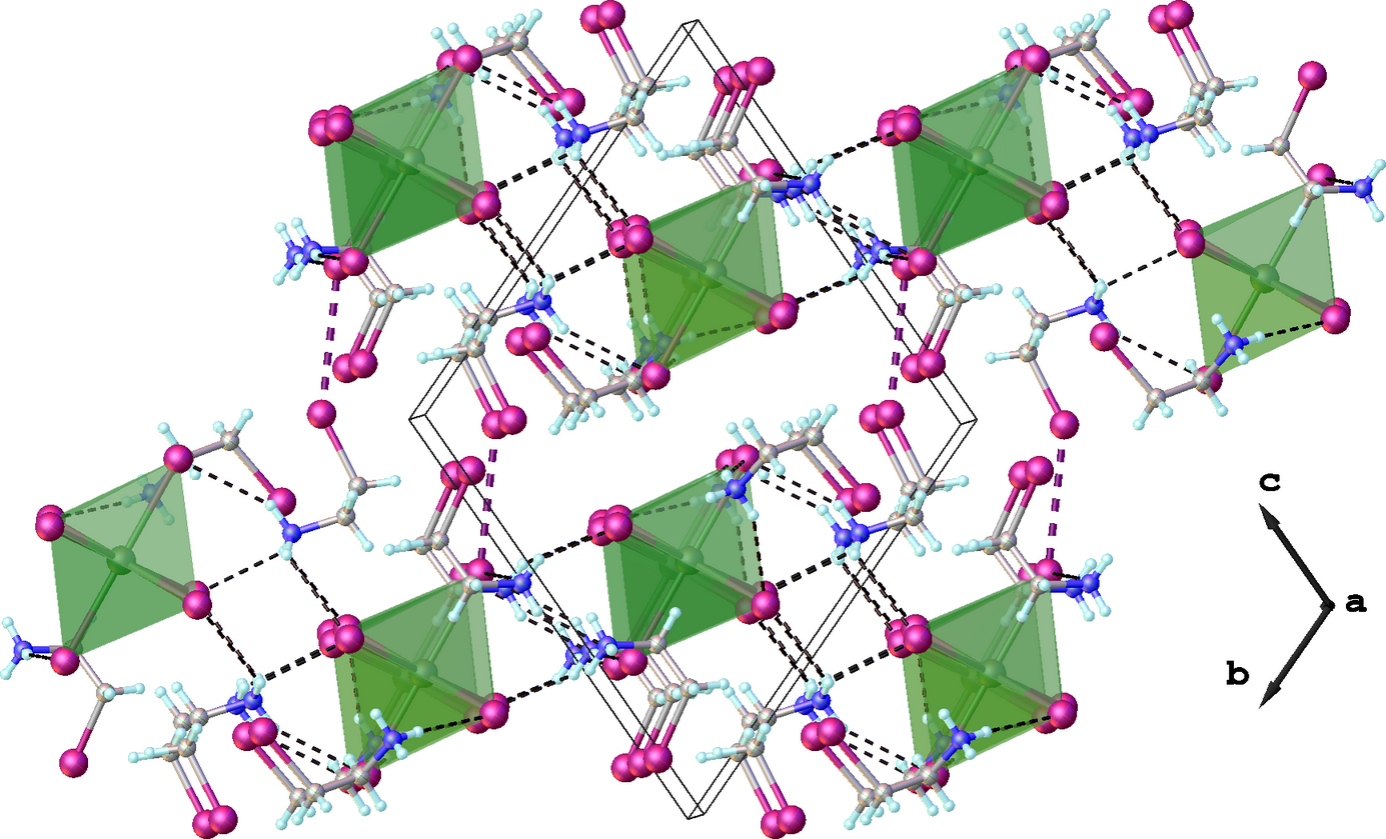
Crystal packing of the title compound plotted along the *a* axis. N—H⋯I hydrogen bonds are drawn as black dashed lines, and weak I⋯I contacts as pink dashed lines.

In the crystal structure, the supra­molecular arrangement is mainly provided by numerous N—H⋯I hydrogen bonds between the ammonium groups of the cations and the I^−^ ligand atoms of the anions (Fig. 2 ▸). All of the I^−^ ligand atoms of the anion, and all of the H atoms of the ammonium NH_3_ groups are involved in these inter­actions, one (N1—H1*C*) in a bifurcated manner (Table 2 ▸). In addition, the distances between the carbon atoms of CH_2_ groups in organic cations and I atoms of coordination octa­hedra range from 3.801 to 3.963 Å suggesting the presence of weak C—H⋯I inter­actions. In addition, a weak I7⋯I3 contact [3.9663 (4) Å] is formed between one iodine atom of 2-iodo­ethyl­ammonium and another iodine atom of the coordination octa­hedron (Fig. 3 ▸). The length of this contact is in the order of the sum of the van der Waals radius of two iodine atoms.

## 4. Hirshfeld surface analysis

**Figure 4:**
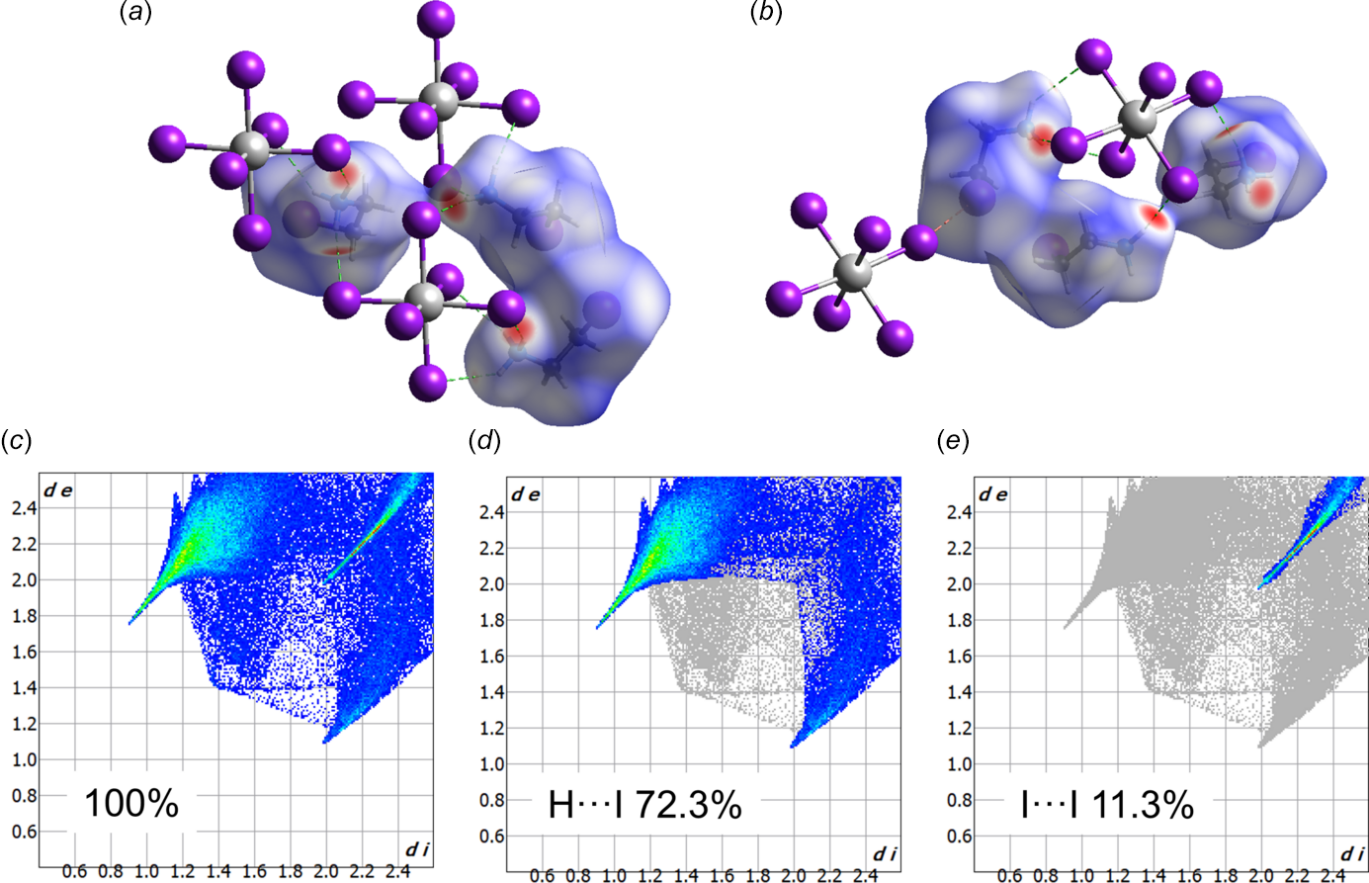
(*a*), (*b*) Hirshfeld surface plotted over a fixed colour scale, which shows the strongest inter­molecular inter­actions in red; (*c*), (*d*), (*e*) two-dimensional fingerprint plots and their percentage contributions.

Weak inter­actions in the structure were additionally analysed by means of a Hirshfeld surface analysis using *CrystalExplorer* (Spackman *et al.*, 2021 ▸). According to the colour code of the calculated Hirshfeld surface (Fig. 4 ▸*a*,*b*), the contacts between atoms with lengths approximately equal to the sum of their van der Waals radii are shown in white, and contacts that are shorter are shown in red, while those that are longer are shown in blue. On the 3-D colour map only N—H⋯I contacts are marked in red, suggesting that these are the strongest inter­actions. The weak I⋯I contact is shown in white, which supports the statement given in the previous section. Two-dimensional fingerprint plots (Fig. 4 ▸*c*–*e*) display the presence of two types of relevant contacts in the structure: H⋯I with 72.3% contribution and I⋯I with 11.3% contribution. The remaining contacts are represented by H⋯H inter­actions.

## 5. Database survey

A search of the Cambridge Structure Database (CSD, version 5.45, updated September 2024; Groom *et al.*. 2016 ▸) revealed that the formation of [Bi_2_I_9_]^3–^ dimers is more common than of isolated [BiI_6_]^3–^ octa­hedra. Some selected examples of crystal structures with discrete [BiI_6_]^3–^ moieties are HUFBAO, which is (MA)_3_[BiI_6_]·3MACl (MA = methyl­ammonium; Zhang *et al.*, 2020 ▸), MAMNEX02, which is (PBA)_4_[BiI_6_]I·H_2_O [(PBA) = C_6_H_5_(CH_2_)_4_NH_3_] (Chen *et al.*, 2021 ▸) and MIJVEK, which is (DPA)_2_[BiI_6_]I_3_ (DPA = C_5_H_16_N_2_; Wang *et al.*, 2023 ▸). The main difference between these structures and the title compound is a mutual arrangement of isolated [BiI_6_]^3–^ inorganic octa­hedra. In the case of HUFBAO, [BiI_6_]^3−^ octa­hedra are stacked along the *ac* plane, in MIJVEK these octa­hedra are located along the *ab* plane and in MAMNEX02 along the *bc* plane. Thus, three examples from the literature can generally be described as structures containing ‘layers’ of inorganic octa­hedra (although these octa­hedra are not bonded to each other), which alternate with an organic component. In the title compound, the inorganic octa­hedra are arranged relative to each other like the vertices of a honeycomb (when viewed along the *a* axis). This arrangement allows for more significant inter­action between the organic and inorganic parts of the structure, resulting in the formation of multiple hydrogen bonds, as described.

## 6. Synthesis and crystallization

Crystals of the title compound have been obtained serendipitously during an intended synthesis of aziridinium (AzrH) bis­muth iodide. (AzrH)_3_[Bi_2_I_9_]·Bi_2_O_3_ (0.1 mmol) was dissolved in 0.5 ml of concentrated HI (57%_wt_). Aziridine (0.1 mol) was dissolved in 1 ml of water and added dropwise to the former solution. Orange crystals formed within 30 minutes, were collected and stored under Paratone(R) oil prior to the diffraction measurement. The formation of (2-IC_2_H_4_NH_3_)_3_[BiI_6_] instead of the target perovskite was established only in the single-crystal X-ray diffraction experiment.

## 7. Refinement

Crystal data, data collection and structure refinement details are summarized in Table 3 ▸. H atoms were placed at calculated positions and refined isotropically with *U*_iso_(H) = 1.2*U*_eq_(C) or *U*_iso_(H) = 1.2*U*_eq_(N). H atoms of secondary CH_2_ groups were refined as riding, while H atoms of NH_3_^+^ groups were refined as rotating.

## Supplementary Material

Crystal structure: contains datablock(s) I. DOI: 10.1107/S2056989025000386/wm5745sup1.cif

Structure factors: contains datablock(s) I. DOI: 10.1107/S2056989025000386/wm5745Isup2.hkl

CCDC reference: 2417294

Additional supporting information:  crystallographic information; 3D view; checkCIF report

## Figures and Tables

**Table 1:** Selected bond lengths (Å)

Bi1—I6	3.0287 (3)	Bi1—I4	3.0786 (3)
Bi1—I3	3.0698 (3)	Bi1—I1	3.1068 (4)
Bi1—I2	3.0733 (3)	Bi1—I5	3.1333 (3)

**Table 2:** Hydrogen-bond geometry (Å, °)

*D*—H⋯*A*	*D*—H	H⋯*A*	*D*⋯*A*	*D*—H⋯*A*
N1—H1*A*⋯I1	0.94	2.77	3.639 (4)	154
N1—H1*B*⋯I5^i^	0.94	3.04	3.735 (4)	132
N1—H1*C*⋯I5	0.94	2.97	3.726 (4)	138
N1—H1*C*⋯I4^i^	0.94	3.08	3.751 (4)	130
N2—H2*A*⋯I2	0.84	2.83	3.660 (4)	170
N2—H2*B*⋯I4^ii^	0.84	2.98	3.716 (4)	148
N2—H2*C*⋯I1^ii^	0.84	3.00	3.730 (4)	146
N3—H3*C*⋯I3	0.85	2.89	3.712 (4)	167
N3—H3*D*⋯I6^iii^	0.85	2.91	3.659 (4)	148
N3—H3*E*⋯I3^iii^	0.85	2.90	3.614 (4)	144

Symmetry codes: (i) 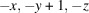; (ii) 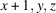; (iii) 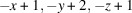.

**Table 3:** Experimental details

Crystal data	
Chemical formula	(C_2_H_7_IN)_3_·[BiI_6_]
*M*_r_	1486.34
Crystal system, space group	Triclinic, *P* 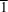
Temperature (K)	100
*a*, *b*, *c* (Å)	8.5014 (2), 12.8233 (3), 13.6364 (3)
α, β, γ (°)	107.689 (2), 107.067 (2), 92.749 (2)
*V* (Å^3^)	1338.49 (6)
*Z*	2
Radiation type	Mo *K*α
μ (mm^−1^)	16.96
Crystal size (mm)	0.19 × 0.1 × 0.05

Data collection	
Diffractometer	XtaLAB Synergy, Dualflex, HyPix
Absorption correction	Analytical (*CrysAlis PRO*; Rigaku OD, 2023 ▸)
*T*_min_, *T*_max_	0.137, 0.506
No. of measured, independent and observed [*I* > 2σ(*I*)] reflections	19499, 6651, 5947
*R*_int_	0.028
(sin θ/λ)_max_ (Å^−1^)	0.712

Refinement	
*R*[*F*^2^ > 2σ(*F*^2^)], *wR*(*F*^2^), *S*	0.025, 0.049, 1.04
No. of reflections	6651
No. of parameters	179
H-atom treatment	H atoms treated by a mixture of independent and constrained refinement
Δρ_max_, Δρ_min_ (e Å^−3^)	2.38, −1.94

Computer programs: *CrysAlis PRO* (Rigaku OD, 2023 ▸), *SHELXT* (Sheldrick, 2015*a* ▸), *SHELXL* (Sheldrick, 2015*b* ▸, *OLEX2* (Dolomanov *et al.*, 2009 ▸) and *publCIF* (Westrip, 2010 ▸).

## supplementary crystallographic information

### Tris(2-iodoethylammonium) hexaiodidobismuthate(III) Crystal data

**Table:**

(C_2_H_7_IN)_3_·[BiI_6_]	*Z* = 2
*M**_r_* = 1486.34	*F*(000) = 1276
Triclinic, *P*1	*D*_x_ = 3.688 Mg m^−^^3^
*a* = 8.5014 (2) Å	Mo *K*α radiation, λ = 0.71073 Å
*b* = 12.8233 (3) Å	Cell parameters from 12539 reflections
*c* = 13.6364 (3) Å	θ = 2.5–30.2°
α = 107.689 (2)°	µ = 16.96 mm^−^^1^
β = 107.067 (2)°	*T* = 100 K
γ = 92.749 (2)°	Prism, clear intense orange
*V* = 1338.49 (6) Å^3^	0.19 × 0.1 × 0.05 mm

### Tris(2-iodoethylammonium) hexaiodidobismuthate(III) Data collection

**Table:**

XtaLAB Synergy, Dualflex, HyPix diffractometer	5947 reflections with *I* > 2σ(*I*)
Detector resolution: 10.0000 pixels mm^-1^	*R*_int_ = 0.028
ω scans	θ_max_ = 30.4°, θ_min_ = 2.5°
Absorption correction: analytical (CrysAlisPro; Rigaku OD, 2023)	*h* = −11→10
*T*_min_ = 0.137, *T*_max_ = 0.506	*k* = −16→17
19499 measured reflections	*l* = −18→18
6651 independent reflections	

### Tris(2-iodoethylammonium) hexaiodidobismuthate(III) Refinement

**Table:**

Refinement on *F*^2^	Hydrogen site location: inferred from neighbouring sites
Least-squares matrix: full	H atoms treated by a mixture of independent and constrained refinement
*R*[*F*^2^ > 2σ(*F*^2^)] = 0.025	*w* = 1/[σ^2^(*F*_o_^2^) + (0.0173*P*)^2^ + 0.4063*P*] where *P* = (*F*_o_^2^ + 2*F*_c_^2^)/3
*wR*(*F*^2^) = 0.049	(Δ/σ)_max_ = 0.001
*S* = 1.04	Δρ_max_ = 2.38 e Å^−^^3^
6651 reflections	Δρ_min_ = −1.94 e Å^−^^3^
179 parameters	Extinction correction: SHELXL (Sheldrick, 2015b), Fc^*^=kFc[1+0.001xFc^2^λ^3^/sin(2θ)]^-1/4^
0 restraints	Extinction coefficient: 0.00014 (3)

### Tris(2-iodoethylammonium) hexaiodidobismuthate(III) Special details

**Table:**

Geometry. All esds (except the esd in the dihedral angle between two l.s. planes) are estimated using the full covariance matrix. The cell esds are taken into account individually in the estimation of esds in distances, angles and torsion angles; correlations between esds in cell parameters are only used when they are defined by crystal symmetry. An approximate (isotropic) treatment of cell esds is used for estimating esds involving l.s. planes.

### Tris(2-iodoethylammonium) hexaiodidobismuthate(III) Fractional atomic coordinates and isotropic or equivalent isotropic displacement parameters (Å^2^)

**Table:**

	*x*	*y*	*z*	*U*_iso_*/*U*_eq_	
Bi1	0.16816 (2)	0.69742 (2)	0.33843 (2)	0.01111 (5)	
I5	0.33222 (3)	0.60094 (2)	0.15861 (2)	0.01361 (7)	
I4	−0.16844 (3)	0.62750 (2)	0.15397 (2)	0.01349 (7)	
I1	0.11528 (3)	0.46673 (2)	0.36362 (2)	0.01363 (7)	
I3	0.19810 (4)	0.92705 (2)	0.31390 (2)	0.01440 (7)	
I2	0.51762 (3)	0.76130 (2)	0.50855 (2)	0.01362 (7)	
I6	0.01073 (3)	0.79056 (3)	0.51288 (2)	0.01571 (7)	
I8	0.58571 (4)	0.31642 (3)	0.11272 (3)	0.01952 (8)	
I7	0.17041 (4)	0.14625 (3)	0.15984 (3)	0.02642 (9)	
I9	0.66397 (5)	1.02469 (3)	0.16011 (3)	0.03205 (9)	
N2	0.6922 (4)	0.5384 (3)	0.3540 (3)	0.0149 (8)	
H2A	0.663 (3)	0.5957 (17)	0.390 (2)	0.018*	
H2B	0.684 (3)	0.5409 (14)	0.2918 (18)	0.018*	
H2C	0.791 (3)	0.5349 (12)	0.386 (2)	0.018*	
N3	0.6590 (5)	0.9734 (4)	0.3921 (3)	0.0219 (10)	
H3C	0.556 (3)	0.9749 (17)	0.3818 (15)	0.026*	
H3D	0.704 (4)	1.034 (2)	0.3922 (16)	0.026*	
H3E	0.704 (4)	0.9647 (14)	0.453 (2)	0.026*	
N1	0.0028 (5)	0.3580 (3)	0.0687 (3)	0.0183 (9)	
H1A	0.031 (4)	0.3599 (4)	0.141 (2)	0.022*	
H1B	−0.086 (3)	0.3978 (13)	0.0535 (17)	0.022*	
H1C	0.095 (3)	0.3901 (12)	0.0579 (18)	0.022*	
C5	0.6821 (6)	0.8794 (4)	0.3028 (4)	0.0204 (11)	
H5A	0.801517	0.884170	0.309359	0.025*	
H5B	0.647553	0.808515	0.310931	0.025*	
C4	0.6282 (6)	0.3321 (4)	0.2813 (4)	0.0180 (10)	
H4A	0.562064	0.268387	0.284755	0.022*	
H4B	0.747215	0.329848	0.316224	0.022*	
C1	−0.0476 (6)	0.2402 (4)	−0.0057 (4)	0.0177 (10)	
H1D	−0.089761	0.239060	−0.081909	0.021*	
H1E	−0.139406	0.205092	0.009941	0.021*	
C3	0.5814 (5)	0.4380 (4)	0.3433 (4)	0.0167 (10)	
H3A	0.464643	0.442694	0.305470	0.020*	
H3B	0.588874	0.436813	0.416807	0.020*	
C2	0.0940 (6)	0.1740 (4)	0.0064 (4)	0.0207 (11)	
H2D	0.190098	0.213498	−0.001433	0.025*	
H2E	0.059806	0.101576	−0.052859	0.025*	
C6	0.5834 (6)	0.8803 (4)	0.1924 (4)	0.0244 (12)	
H6A	0.464389	0.877912	0.186693	0.029*	
H6B	0.594239	0.813096	0.136611	0.029*	

### Tris(2-iodoethylammonium) hexaiodidobismuthate(III) Atomic displacement parameters (Å^2^)

**Table:**

	*U*^11^	*U*^22^	*U*^33^	*U*^12^	*U*^13^	*U*^23^
Bi1	0.01029 (9)	0.01076 (9)	0.01174 (9)	0.00051 (6)	0.00391 (7)	0.00285 (7)
I5	0.01205 (14)	0.01543 (16)	0.01372 (15)	0.00326 (11)	0.00523 (12)	0.00415 (12)
I4	0.01107 (14)	0.01490 (15)	0.01383 (15)	0.00077 (11)	0.00348 (11)	0.00456 (12)
I1	0.01375 (14)	0.01310 (15)	0.01452 (15)	0.00132 (11)	0.00420 (12)	0.00576 (12)
I3	0.01689 (15)	0.01120 (15)	0.01465 (15)	0.00080 (11)	0.00440 (12)	0.00454 (12)
I2	0.01114 (14)	0.01473 (15)	0.01353 (15)	0.00037 (11)	0.00297 (11)	0.00390 (12)
I6	0.01468 (15)	0.01651 (16)	0.01551 (16)	0.00063 (11)	0.00765 (12)	0.00232 (12)
I8	0.02162 (16)	0.01834 (17)	0.01683 (16)	−0.00061 (12)	0.00818 (13)	0.00205 (13)
I7	0.02723 (18)	0.0288 (2)	0.0284 (2)	0.00564 (14)	0.00640 (15)	0.01908 (16)
I9	0.0554 (2)	0.0230 (2)	0.02364 (19)	0.00998 (17)	0.01751 (17)	0.01078 (15)
N2	0.0132 (19)	0.014 (2)	0.015 (2)	0.0011 (15)	0.0032 (16)	0.0023 (16)
N3	0.021 (2)	0.024 (2)	0.019 (2)	−0.0036 (17)	0.0082 (18)	0.0047 (18)
N1	0.025 (2)	0.013 (2)	0.018 (2)	0.0042 (16)	0.0068 (17)	0.0050 (17)
C5	0.030 (3)	0.011 (2)	0.020 (3)	0.003 (2)	0.009 (2)	0.004 (2)
C4	0.018 (2)	0.015 (3)	0.021 (3)	−0.0007 (19)	0.004 (2)	0.008 (2)
C1	0.015 (2)	0.014 (2)	0.018 (3)	−0.0010 (18)	0.0014 (19)	0.0018 (19)
C3	0.013 (2)	0.024 (3)	0.013 (2)	−0.0011 (19)	0.0048 (19)	0.007 (2)
C2	0.019 (2)	0.021 (3)	0.022 (3)	0.004 (2)	0.007 (2)	0.008 (2)
C6	0.031 (3)	0.021 (3)	0.018 (3)	0.000 (2)	0.008 (2)	0.002 (2)

### Tris(2-iodoethylammonium) hexaiodidobismuthate(III) Geometric parameters (Å, º)

**Table:**

Bi1—I6	3.0287 (3)	N1—H1B	0.94 (3)
Bi1—I3	3.0698 (3)	N1—H1C	0.94 (3)
Bi1—I2	3.0733 (3)	N1—C1	1.498 (6)
Bi1—I4	3.0786 (3)	C5—H5A	0.9900
Bi1—I1	3.1068 (4)	C5—H5B	0.9900
Bi1—I5	3.1333 (3)	C5—C6	1.496 (7)
I8—C4	2.163 (5)	C4—H4A	0.9900
I7—C2	2.145 (5)	C4—H4B	0.9900
I9—C6	2.155 (5)	C4—C3	1.506 (7)
N2—H2A	0.84 (2)	C1—H1D	0.9900
N2—H2B	0.84 (2)	C1—H1E	0.9900
N2—H2C	0.84 (2)	C1—C2	1.505 (6)
N2—C3	1.502 (6)	C3—H3A	0.9900
N3—H3C	0.85 (3)	C3—H3B	0.9900
N3—H3D	0.85 (3)	C2—H2D	0.9900
N3—H3E	0.85 (3)	C2—H2E	0.9900
N3—C5	1.499 (6)	C6—H6A	0.9900
N1—H1A	0.94 (3)	C6—H6B	0.9900
			
I4—Bi1—I5	86.834 (9)	H5A—C5—H5B	107.9
I4—Bi1—I1	87.370 (9)	C6—C5—N3	112.2 (4)
I1—Bi1—I5	91.043 (9)	C6—C5—H5A	109.2
I3—Bi1—I5	91.561 (9)	C6—C5—H5B	109.2
I3—Bi1—I4	90.279 (9)	I8—C4—H4A	109.2
I3—Bi1—I1	176.391 (9)	I8—C4—H4B	109.2
I3—Bi1—I2	89.461 (9)	H4A—C4—H4B	107.9
I2—Bi1—I5	88.607 (9)	C3—C4—I8	112.2 (3)
I2—Bi1—I4	175.425 (10)	C3—C4—H4A	109.2
I2—Bi1—I1	93.101 (9)	C3—C4—H4B	109.2
I6—Bi1—I5	179.779 (10)	N1—C1—H1D	109.1
I6—Bi1—I4	93.367 (9)	N1—C1—H1E	109.1
I6—Bi1—I1	88.876 (9)	N1—C1—C2	112.5 (4)
I6—Bi1—I3	88.529 (9)	H1D—C1—H1E	107.8
I6—Bi1—I2	91.192 (9)	C2—C1—H1D	109.1
H2A—N2—H2B	109.5	C2—C1—H1E	109.1
H2A—N2—H2C	109.5	N2—C3—C4	112.2 (4)
H2B—N2—H2C	109.5	N2—C3—H3A	109.2
C3—N2—H2A	109.5	N2—C3—H3B	109.2
C3—N2—H2B	109.5	C4—C3—H3A	109.2
C3—N2—H2C	109.5	C4—C3—H3B	109.2
H3C—N3—H3D	109.5	H3A—C3—H3B	107.9
H3C—N3—H3E	109.5	I7—C2—H2D	109.0
H3D—N3—H3E	109.5	I7—C2—H2E	109.0
C5—N3—H3C	109.5	C1—C2—I7	113.0 (3)
C5—N3—H3D	109.5	C1—C2—H2D	109.0
C5—N3—H3E	109.5	C1—C2—H2E	109.0
H1A—N1—H1B	109.5	H2D—C2—H2E	107.8
H1A—N1—H1C	109.5	I9—C6—H6A	109.1
H1B—N1—H1C	109.5	I9—C6—H6B	109.1
C1—N1—H1A	109.5	C5—C6—I9	112.3 (3)
C1—N1—H1B	109.5	C5—C6—H6A	109.1
C1—N1—H1C	109.5	C5—C6—H6B	109.1
N3—C5—H5A	109.2	H6A—C6—H6B	107.9
N3—C5—H5B	109.2		
			
I8—C4—C3—N2	−66.2 (4)	N1—C1—C2—I7	68.6 (5)
N3—C5—C6—I9	64.1 (5)		

### Tris(2-iodoethylammonium) hexaiodidobismuthate(III) Hydrogen-bond geometry (Å, º)

**Table:**

*D*—H···*A*	*D*—H	H···*A*	*D*···*A*	*D*—H···*A*
N1—H1*A*···I1	0.94	2.77	3.639 (4)	154
N1—H1*B*···I5^i^	0.94	3.04	3.735 (4)	132
N1—H1*C*···I5	0.94	2.97	3.726 (4)	138
N1—H1*C*···I4^i^	0.94	3.08	3.751 (4)	130
N2—H2*A*···I2	0.84	2.83	3.660 (4)	170
N2—H2*B*···I4^ii^	0.84	2.98	3.716 (4)	148
N2—H2*C*···I1^ii^	0.84	3.00	3.730 (4)	146
N3—H3*C*···I3	0.85	2.89	3.712 (4)	167
N3—H3*D*···I6^iii^	0.85	2.91	3.659 (4)	148
N3—H3*E*···I3^iii^	0.85	2.90	3.614 (4)	144

Symmetry codes: (i) −*x*, −*y*+1, −*z*; (ii) *x*+1, *y*, *z*; (iii) −*x*+1, −*y*+2, −*z*+1.
